# Selective enrichment of the raw milk microbiota in cheese production: Concept of a natural adjunct milk culture

**DOI:** 10.3389/fmicb.2023.1154508

**Published:** 2023-04-26

**Authors:** Luca Bettera, Matthias Dreier, Remo S. Schmidt, Monica Gatti, Hélène Berthoud, Hans-Peter Bachmann

**Affiliations:** ^1^Department of Food and Drug, University of Parma, Parma, Italy; ^2^Agroscope, Bern, Switzerland

**Keywords:** cheese quality, raw milk cheese, natural milk culture, NSLAB, fermented food, microbial communities

## Abstract

In cheese production, microorganisms are usually added at the beginning of the process as primary starters to drive curd acidification, while secondary microorganisms, with other pro-technological features important for cheese ripening, are added as selected cultures. This research aimed to investigate the possibilities of influencing and selecting the raw milk microbiota using artisanal traditional methods, providing a simple method to produce a natural supplementary culture. We investigated the production of an enriched raw milk whey culture (eRWC), a natural adjunct microbial culture produced from mixing an enriched raw milk (eRM) with a natural whey culture (NWC). The raw milk was enriched by spontaneous fermentation for 21 d at 10°C. Three milk enrichment protocols were tested: heat treatment before incubation, heat treatment plus salt addition, and no treatment. The eRMs were then co-fermented with NWC (ratio of 1:10) at 38°C for 6 h (young eRWC) and 22 h (old eRWC). Microbial diversity during cultures’ preparation was evaluated through the determination of colony forming units on selective growth media, and next-generation sequencing (16S rRNA gene amplicon sequencing). The enrichment step increased the streptococci and lactobacilli but reduced microbial richness and diversity of the eRMs. Although the lactic acid bacteria viable count was not significantly different between the eRWCs, they harbored higher microbial richness and diversity than NWC. Natural adjunct cultures were then tested in cheese making trials, following the microbial development, and assessing the chemical quality of the 120 d ripened cheeses. The use of eRWCs slowed the curd acidification in the first hours of cheese making but the pH 24 h after production settled to equal values for all the cheeses. Although the use of diverse eRWCs contributed to having a richer and more diverse microbiota in the early stages of cheese making, their effect decreased over time during ripening, showing an inferior effect to the raw milk microbiota. Even if more research is needed, the optimization of such a tool could be an alternative to the practice of isolating, geno-pheno-typing, and formulating mixed-defined-strain adjunct cultures that require knowledge and facilities not always available for artisanal cheese makers.

## 1. Introduction

Microorganisms are most often intentionally applied in cheese making. They are added at the beginning of the process as primary starters, where they drive curd acidification by metabolizing milk lactose into lactic acid. Further, secondary microorganisms have features important for cheese ripening (e.g., eye formation, rind modification, flavor enhancement). Additionally, in cheese varieties made from raw milk, an autochthonous non-starter microbiota of milk origin is present in the ripened cheeses. These microbes, mostly lactic acid bacteria (LAB), contribute to the formation of the cheese flavor and texture. This increased microbial diversity can change the characteristics of the cheese due to the presence of other metabolic pathways. On the one hand, this can lead to authenticity and added value, but on the other hand, it can also lead to off-flavors and/or texture deficiencies. Reproducibility of consistent flavor and quality when using raw milk is difficult because the composition of the microbiota in the milk varies. The growth dynamics of strains from raw milk during production and maturation are difficult to predict.

Artisanal natural cultures represent a practical tool for cheesemakers to influence the fermentation process while keeping a high biodiversity linked to the terroir of production. These cultures can be maintained in-house by backslopping (i.e., use of an old batch of a fermented product to inoculate a new one). Natural cultures have an undefined strain composition, although the application of selective pressure (heat treatment, incubation temperature, low pH) favors the dominance of desired LAB (Parente et al., 2017; Powell et al., 2022). These cultures are produced either from milk or whey (Parente, 2006), and they are required by the standards of identity of several traditional protected designation of origin [PDO; (EP and Council of EU, 2012)] cheese types because a strict relationship is believed to exist between their use, the cheese quality and the territory of production.

Natural milk cultures (NMC) are used in the production of several traditional cheeses, such as Argentinian cheese (Reinheimer et al., 1997), Montasio PDO (Marino et al., 2003, 2008; Carraro et al., 2011), Asiago PDO (Gobbetti et al., 2018), and Mozzarella TSG [Traditional Speciality Guaranteed; (EP and Council of EU, 2012)]. These cultures are produced starting from raw milk, which is thermized (60°C–65°C, 10–30 min), and incubated at 42°C–45°C until a titratable acidity of 0.4%–0.6% lactic acid is reached (Parente, 2006). Dominating species of NMC are *Streptococcus thermophilus*, *Lactobacillus delbrueckii* subsp. *lactis* and *Lactobacillus helveticus*, but other thermophilic and mesophilic LAB (e.g., *Streptococcus gallolyticus* subsp. *macedonicus*, enterococci) may be present as co-dominant or sub-dominant species (Zotta et al., 2022).

Natural whey cultures (NWC) are produced by incubating whey drained from the cheese (commonly referred as “sweet” whey) in conditions that favour the selection of desirable LAB. The incubating temperature varies according to the cheese variety, and can be controlled or uncontrolled, i.e., spontaneously decreasing from the initial temperature to room temperature. NWC are widely used for the production of Italian cheese varieties, including Mozzarella di Bufala Campana (De Filippis et al., 2014), Caciocavallo Silano (Ercolini et al., 2008), Nostrano Valtrompia, Provolone (Gobbetti et al., 2018), but also French [e.g., Comté PDO, Rocamadour PDO (Demarigny et al., 2006), Picodon PDO (Yann and Pauline, 2014)] and Swiss varieties (L’Etivaz PDO, Berner Alp- und Hobelkäse PDO, Le Gruyère PDO). The characteristics of NWC used for the production of Grana Padano and Parmigiano Reggiano have been reviewed by Gatti et al. (2014). The amount of culture added to the vat milk varies between 2.7% and 3.5% depending on the value of the titratable acidity determined on the production day. The main drivers for the selection of wanted LAB are the high incubation temperature, the low pH at the end of incubation and the backslopping process itself, as favorable LAB are present at the beginning of the inoculation. These conditions lead to the dominance of the aciduric and thermophilic LAB that reach a concentration ranging from 7.7 to 9.9 log CFU/mL. Dominant LAB species of Grana Padano and Parmigiano Reggiano NWC are *L. helveticus, L. delbrueckii* subsp. *lactis*, and less frequently *Limosilactobacillus fermentum* and *S. thermophilus* (Gatti et al., 2014). The NWC used for Gruyère cheese making is also composed of thermophilic LAB, since the initial whey is incubated at 38°C for 20 h after having reached the curd cooking temperature of 54°C–59°C and a further thermization to about 60°C–63°C (Moser et al., 2018).

However, to the authors’ knowledge, natural cultures are exclusively used as primary cultures. Commercial secondary cultures with non-starter lactic acid bacteria (NSLAB) strains (also called adjunct cultures) are already available on the market, but this may not be a suitable solution to ensure broad species diversity (Gobbetti et al., 2015). Moreover, these mixed- or defined-strain cultures are usually maintained and cultivated in the laboratory and cheese makers may not be able to maintain them using traditional methods (Powell et al., 2022).

In this work, we wanted to investigate the possibilities of influencing and selecting the microbiota of raw milk using artisanal traditional methods, with the aim of providing a simple method for the production of a natural supplementary culture for artisanal cheese makers. We investigated the production of an enriched raw milk whey culture (eRWC), a natural adjunct microbial culture produced from co-fermenting an enriched raw milk (eRM) with a natural whey culture (NWC). The eRWC was produced in triplicate testing different protocols and analyzed for the viable microbial profile with classical microbiology, and for its microbiota through next-generation sequencing (NGS). Afterwards, the eRWC were tested in two trials as adjunct cultures for the production of Vacherin Fribourgeois PDO, evaluating the microbial evolution and the cheese chemical features up to 120 d of ripening.

## 2. Materials and methods

### 2.1. Natural cultures production

The raw milk and the sweet whey (deriving from Gruyère cheese production) were provided by the Agricultural Institute in Grangeneuve (Switzerland) within the framework of the Center of Excellence for Raw Milk Products. The samples were delivered to the laboratory for analysis on the same morning in refrigerated condition.

**Figure 1:**
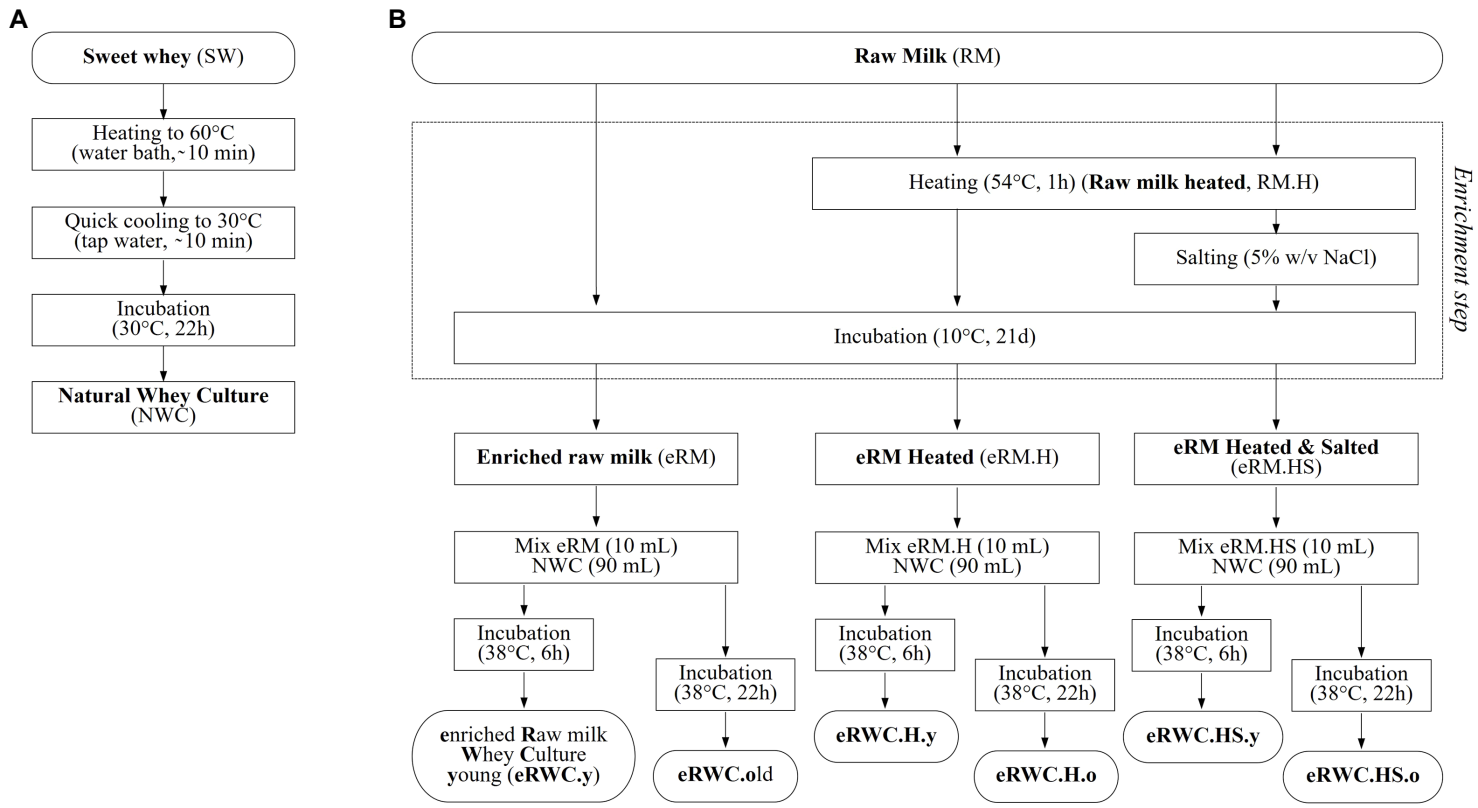
Natural adjunct culture production flow chart: **(A)** natural whey culture; **(B)** raw milk enrichment step and final enriched raw milk whey culture production. Reference for the samples’ abbreviation.

The eRWCs production process is schematized in Figure 1. Briefly, the raw milk (RM) was enriched by spontaneous fermentation for 21 d at 10°C. Three milk enrichment protocols were tested: heat treatment (1 h at 54°C) before incubation (eRM.H), heat treatment plus salt addition (1 h at 54°C, 5% w/v NaCl; eRM.HS), and no treatment (eRM). After mixing 1 part of eRM with 9 of NWC, the cultures were further incubated at 38°C for 6 h and 22 h to obtain the so-called “young” (y) and “old” (o) cultures, respectively, resulting in a total of 6 final cultures: (1) eRWC.y; (2) eRWC.o; (3) eRWC.H.y; (4) eRWC.H.o; (5) eRWC.HS.y; (6) eRWC.HS.o.

With the purpose to give a basic description of the adjunct cultures’ aroma, at the end of the incubation period the samples were smelled by the operators in order to evaluate the presence or absence of four aroma descriptors: expired milk (off-flavor), fresh milk, acidic-fresh yoghurt, and cheese flavor.

The pH-value of the samples was measured using a pH electrode (Knick Elektronische Messgeräte GmbH & Co. KG, Berlin, Germany).

The titratable acidity was determined by titration of 10 mL of sample with NaOH (0.25 N) using phenolphthalein (2%) as indicator. Results were expressed in Soxhlet-Henkel degrees (°SH) (Deutscher Normenausschuss, 1970).

### 2.2. Microbiological analysis

For the microbiological analysis, 10 g of cheese core samples were homogenized in 90 mL of peptone-buffered saline water using a Stomacher (Masticator, IUL Instruments, Königswinter, Germany).

Homogenized cheese and the liquid samples were diluted 10-fold in peptone-buffered saline water for the viable counts of different microbial groups. Supplementary Table S1 reports the agar growth media used and their incubation condition. Each sample was plated in duplicate; results are expressed as log_10_ of colony forming units (CFU) per mL (log CFU/mL). For plates without colonies due to the detection limit or excessive sample dilution, microbial counts were either expressed as zero (i.e., absent in 1 mL) or just below the lowest dilution analyzed (e.g., absent in the 10^1^ dilution = 9 CFU/mL).

The count of *Clostridium* spores was performed by the most probable number technique (MPN), using the BY liquid growth medium (Bryant and Burkey, 1956). The sample (1 mL) of sample was mixed with 10 mL of medium, and then heat treated in a water bath at 80°C for 10 min to kill the cells in the vegetative form. The glass tubes containing a glass bell as gas formation indicator, were incubated at 37°C for 7 d. The MPN/mL was calculated according to the McGrady tables for three replicates. The following controls were used: negative control using sterile milk, and two positive controls using sterile milk inoculated with *Clostridium tyrobutyricum* FAM22553 (5 MPN/mL) and *Clostridium sporogenes* FAM1752 (5 MPN/mL) (Agroscope Microbial Collection).

The cheese making samples (i.e., vat milk at day 0, cheese at day 1, cheese at day 60, and cheese at day 120) were analyzed By an external laboratory for The absence of listeria, salmonella, coagulase positive staphylococci and *Escherichia coli*.

### 2.3. Next-generation sequencing

The DNA extraction of 120 d ripened cheese core, and subsequent 16S rRNA gene amplicon sequencing and analysis were performed following (Dreier et al., 2022).

For the DNA extraction of liquid samples, a pellet was obtained by centrifuging 1 mL of sample at 16,000 × g for 10 min and discarding the supernatant. Then, 600 μL of guanidinium chloride 8 M were added and centrifugated at 16,000 × g for 10 min (this step was avoided for RM samples). After another supernatant removal, 1 mL of guanidium chloride 4 M were added and centrifugated for 5 min at 8,000 × g. After another supernatant removal, 400 μL of G2 buffer solution (EZ1 DNA Tissue kit, Qiagen, Hilden, Germany) were added and the whole sample was transferred to a 0.5 mL skirted tubes containing 100 mg 0.1 mm low binding zirconium beads (OPS Diagnostics, Lebanon, NJ, United States) and shaken for 1 min in a bead ruptor (Omni International Inc., Kennesaw, GA, United States). The tube was centrifugated at 16,000 × g for 10 min; after that, 200 μL of the supernatant were transferred to a tube with 10 μL of proteinase K (Qiagen) and incubated for 1 h at 56°C. Cell lysates were then processed by the BioRobot® EZ1 workstation (Qiagen).

The 16S rRNA gene amplicon sequencing and data analysis were performed as described by Dreier et al. (2022). The amplification was carried out as follows: 98°C for 30 s, followed by 18–35 cycles of 98°C for 10 s, 55°C for 20 s, and 72°C for 30 s, and a final elongation 72°C for 5 min. Sequencing was carried out on an IonTorrent Ion GeneStudio^™^ S5 System instrument (Thermo Fisher Scientific).

The raw sequences were primer trimmed and quality filtered in DADA2 (Callahan et al., 2016). Amplicon sequence variances (ASVs) were obtained in DADA2 with the parameter POOL = “pseudo.” Taxonomic annotation was performed using DAIRYdb v1.2.4 (Meola et al., 2019) with IDTAXA (Murali et al., 2018). Biostatistical analyses were done using the packages PHYLOSEQ (McMurdie and Holmes, 2013) and vegan (Oksanen, 2022) in R v4.2.2 (R Core Team, 2022). Alpha-diversity indices were calculated to measure microbial richness and diversity. Beta-diversity was measured calculating the Bray–Curtis dissimilarity, the statistical significance was verified with a permutational multivariate analysis of variance (PERMANOVA) model.

### 2.4. Cheese production and quality analysis

**Figure 2:**
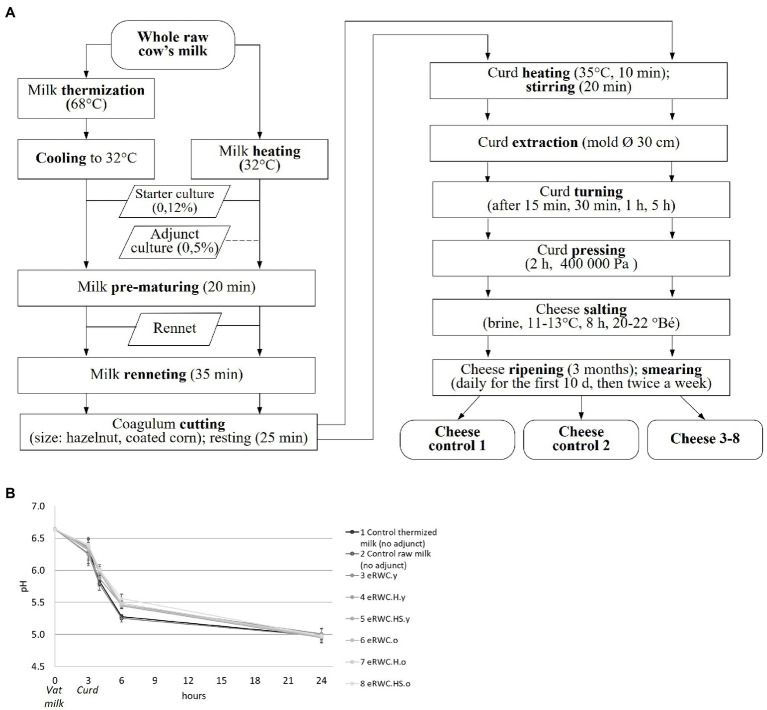
**(A)** Vacherin Fribourgeois PDO cheese making flow chart. **(B)** Acidification curve during the cheese making; the adjunct culture added in each trial is reported in the legend. Refer to Figure 1 for samples’ abbreviation.

Eight cheeses were produced following the Vacherin Fribourgeois PDO technology (Figure 2). The trials were replicated in two consequent days in the pilot plant facility at Agroscope (Liebefeld, Switzerland). Two controls, one with raw milk and the other with thermized milk, were produced using only the starter culture. A starter culture commonly used in Vacherin Fribourgeois PDO production (*Lactococcus lactis* and *Leuconostoc mesenteroides*; Liebefeld Kulturen AG) was also added at the proportion of 0.12% to the vat milk. The natural adjunct cultures were added at the proportion of 0.5% to the vat milk. The pH-value of the cheeses was determined using a pH electrode.

#### 2.4.1. Volatile carboxylic acids

Volatile carboxylic acids were analyzed in the 120 d ripened cheeses using a Hewlett Packard HP 6890 gas chromatograph (Agilent Technologies, Basel, Switzerland) as described by Fröhlich-Wyder et al. (2013).

#### 2.4.2. Biogenic amines

Biogenic amines were analyzed in the 120 d ripened cheeses as described by Ascone et al. (2017) using a UPLC system (UltiMate 3000 RS; Thermo Fisher Scientific) equipped with a C18 column (Accucore C18: 2.6 mm, 150 _ 4.6 mm; Thermo Fisher Scientific). All measurements were carried out in duplicate.

#### 2.4.3. Free amino acids and di- and tripeptides

Total free amino acids and di- and tripeptides were analyzed in the 120 d ripened cheeses with the ophthaldialdehyde (OPA) method (Egger et al., 2019). Briefly, the samples were diluted 10-fold, prior to precipitation with perchloric acid (0.5 mol/L), and then derivatized with OPA in the presence of 2-mercapto-ethansulfonic acid. The produced 1-alkylthio-2-alkylisoindol compound was measured at 340 nm. To calculate the results, a standard curve based on glutamic acid was used.

#### 2.4.4. Proteolysis

The extent of proteolysis in the 120 d ripened cheese was measured by analyzing the following compounds: total nitrogen (TN), water-soluble nitrogen (WSN) and non-protein nitrogen (NPN) according to Kjeldahl (Collomb et al., 1990).

#### 2.4.5. Moisture and fat content

Cheese samples at 1 d and 120 d of ripening were analyzed for moisture, dry matter (IDF, 1982), fat (IDF, 1987), and fat in dry matter (FDM) using common standard methods.

#### 2.4.6. Lactic acid, citric acid, and L-leucine aminopeptidase

Cheese samples at 1 d and 120 d of ripening were analyzed for lactic acid and citric acid concentration and L-leucine aminopeptidase (LAP) activity. For the determination of lactate, 1.25 g of cheese were homogenized in 50 mL of water using an OmniPrep Multi-Sample Homogenizer (Omni International, Kennesaw, United States). For the determination of citrate, 5 g of cheese were used. The homogenates were then incubated at 2°C for 20 min. Particles and fat were removed by filtration. The concentration of D- and L-lactate, and citrate in the filtrates was determined using commercial enzymatic assay kits (R-Biopharm AG, Murten, Switzerland). L-leucine-aminopeptidase (LAP) activity was determined using a colorimetric assay with L-leucine-4-nitroanilide as substrate. For the assays, 60 μL cheese filtrate (1.25 g cheese sample homogenized in 50 mL water and filtered) and 250 μL of L-leucine-4-nitroanilide (final concentration: 0.995 mmol/L) in phosphate buffer containing 2 mmol/L Mg^2+^ (pH = 7.4) were mixed in a microtiter plate. Enzyme activity was calculated based on the micromolar extinction coefficient of 4-nitroaniline measured by a SpectraMax ABS plus plate reader (Molecular Devices) after 2 h of incubation using the SoftMax Pro software (Molecular Devices).

### 2.5. Statistical analysis

A statistical analysis of the microbiological results was conducted to evaluate the influence of the enrichment step in changing the microbial composition. RM, eRMs, NWC, and eRWCs were available in triplicate. All the results reported in the text are expressed as mean ± standard deviation. The comparisons RM-eRM, RM.H-eRM.H and RM.H-eRM.HS were done with a Student’s t-test (*α* = 0.05) when the variances were homogeneous (F-test; α = 0.05); a Welch’s *t*-test (*α* = 0.05) was instead applied when the variances were heterogeneous. The difference between the three eRMs and between eRWCs and NWC were instead analyzed with an ANOVA model (*α* = 0.05). In this case, the homogeneity of variances was previously tested with Bartlett’s test (*α* = 0.05); if the ANOVA showed significant differences, a multiple comparisons Tukey’s HSD test was applied. If the ANOVA assumptions were not meet, non-parametric Kruscal–Wallis (*α* = 0.05) and Dunn’s *post hoc* tests were applied. The statistical analysis was done using the packages “stats,” “agricolae” (de Mendiburu, 2021) and “FSA” (Derek et al., 2022) in the R environment (R Core Team, 2022).

Microbiological features (*n* = 14) of vat milk and cheese after 60 d, and both chemical (varying *n*, range 7–29) and microbiological (*n* = 14) features of cheeses after 1 and 120 d were subjected to principal component analysis (PCA). The analysis was done in the R environment using the packages “FactoMineR” (Le et al., 2008) and “factoextra” for the results plotting (Kassambra and Mundt, 2020).

## 3. Results

### 3.1. Culture production

The enriched raw milk (eRMs) underwent phase separation at the end of the 21 d long incubation period. As the samples were left unagitated during incubation, fat accumulated at the top due to spontaneous creaming, while a protein coagulum deposited at the bottom. For eRM.HS, however, we did not observe protein coagulation. All the final eRWCs reached a pH below 4.5 (Table 1). The extended incubation to produce the “old” cultures led to further acidification, with pH values reaching a range of 3.8–3.9.

**Table 1:** Acidity and odor evaluation of RM, RM.H, eRMs, NWC, SW, and eRWCs.

Sample (*n* = 3)	Acidity		Odor evaluation											
	pH	°SH/10 mL	Expired milk (Ooff-flavor)			Fresh milk			Acidic-fresh yoghurt			Cheese flavor		
			Replicates											
			1	2	3	1	2	3	1	2	3	1	2	3
RM	6.6 ± 0	7.3 ± 0.6	–	–	–	–	–	–	–	–	–	–	–	–
RM.H	6.6 ± 0	7.5 ± 0.5	–	–	–	–	–	–	–	–	–	–	–	–
eRM	4.7 ± 0.6	32.7 ± 7.1	✓	✗	✗	✗	✗	✗	✗	✗	✗	✗	✓	✓
eRM.H	6.2 ± 0.2	13 ± 2	✓	✗	✗	✗	✗	✗	✗	✓	✓	✗	✗	✓
eRM.HS	6.4 ± 0.1	10.5 ± 0.9	✗	✗	✗	✓	✓	✓	✗	✗	✗	✗	✗	✗
eRWC.y	4.2 ± 0.1	21 ± 2	✓	✗	✗	✗	✗	✗	✗	✓	✓	✗	✗	✗
eRWC.H.y	4.2 ± 0.2	21 ± 3.5	✗	✗	✗	✗	✗	✗	✓	✓	✓	✗	✗	✗
eRWC.HS.y	4.2 ± 0.2	21.7 ± 4	✗	✗	✗	✗	✗	✗	✓	✓	✓	✗	✗	✗
eRWC.o	3.9 ± 0.4	32.7 ± 11.5	✓	✗	✗	✗	✗	✗	✗	✓	✓	✗	✗	✗
eRWC.H.o	3.9 ± 0.4	33 ± 12.1	✗	✗	✗	✗	✗	✗	✓	✓	✓	✓	✗	✗
eRWC.HS.o	3.8 ± 0.4	32.3 ± 10.3	✗	✗	✗	✗	✗	✗	✓	✓	✓	✗	✗	✗
SW	6.5 ± 0.1	5.3 ± 0.6	–	–	–	–	–	–	–	–	–	–	–	–
NWC	4.6 ± 0.5	16.7 ± 3.2	✗	✗	✗	✗	✗	✗	✓	✓	✓	✗	✗	✗

Refer to Figure 1 for samples’ abbreviation (✓ = perceived; ✗ = not perceived; – = not evaluated).

The raw milk treatment influenced the odor of the eRMs and of the final cultures ready for addition to vat milk at the end of their respective incubation periods. For the eRMs, the outcomes differed for the three setups: two out of three replicates of eRM developed a cheese flavor; two out of three replicates of eRM.H developed an acidic-fresh yoghurt-like flavor; while all the three eRM.HS replicates kept a flavor similar to fresh milk (Table 1). The final eRWC cultures had an acidic-fresh yoghurt flavor like the NWC, with two exceptions, where off-flavors were detected.

#### 3.1.1. Microbiological analysis

The RM, RM.H, eRMs, NWC and eRWCs samples were analyzed for the viable count of the microbial groups listed in Supplementary Table S1. All the results are presented in Table 2. The hygienic quality of the RM was satisfactory, having an average value of total aerobic mesophilic (TAM) within European and Swiss legal criteria (EC, 2004; EDI, 2017), equal to 4.9 ± 0.9 log CFU/mL (mean ± SD). When the RM was heat treated, all the bacterial groups decreased in concentration (TAM = 2.1 ± 0.2 log CFU/mL).

**Table 2:** Viable microbial counts results (mean ± SD).

Sample (*n* = 3)	Aerobic mesophile	Streptococci	Lactobacilli	FH lactobacilli	Enterococci	Yeasts and molds	Staphylococci	Enterobacteriaceae
A								
RM	4.9 ± 0.9	4.3 ± 0.9	3.1 ± 0.6	1 ± 0.1	2.3 ± 0.3	1.1 ± 0.3	4.3 ± 0.3	1.7 ± 0.3
RM.H	2.1 ± 0.2	1.4 ± 0.2	0.8 ± 0.3	0 ± 0	0.7 ± 0.3	0 ± 0	1 ± 0	0.3 ± 0.3
eRM	9.2 ± 0*^a^	9.1 ± 0.2*^a^	9.1 ± 0.2*^a^	3.6 ± 0.6*^a^	6.8 ± 1.3*^a^	4.6 ± 0.4*^a^	6.8 ± 0.5*^a^	6.5 ± 0.8*^a^
eRM.H	7.9 ± 0.4*^b^	7.7 ± 0.6*^ab^	7.6 ± 1.2*^a^	3.1 ± 0.7*^a^	7 ± 0.7*^a^	3.8 ± 1.4*^ab^	6.6 ± 1*^a^	6.8 ± 1*^a^
eRM.HS	6.6 ± 0.4*^c^	6.1 ± 1.2*^b^	4.5 ± 0.8*^b^	0.4 ± 0.6^b^	4.7 ± 0.7*^a^	1.3 ± 1.5^b^	6 ± 1.1*^a^	3.7 ± 0.4*^b^
B								
NWC	8.5 ± 0.7	7.8 ± 1.1	7.7 ± 1.3	2.3 ± 1.2	2.8 ± 0.6 ^b^	1.2 ± 2^b^	2.3 ± 0.4^b^	0.4 ± 0.7^b^
eRWC.y	8 ± 0.7	7.5 ± 0.7	7.9 ± 0.2	2.1 ± 0.4	5.8 ± 1.9 ^ab^	4.1 ± 0.8^ab^	4 ± 0.6^ab^	3.2 ± 1.1^ab^
eRWC.o	7.3 ± 1	6.5 ± 0.2	7.4 ± 1.2	1.7 ± 0.5	5.5 ± 0.8^ab^	5.7 ± 1.2^a^	3.2 ± 1.3^ab^	4.8 ± 1.8^a^
eRWC.H.y	8.3 ± 0.8	7.7 ± 1.1	7.8 ± 0.9	2.3 ± 1.2	5.9 ± 0.9^ab^	4.4 ± 1.1^ab^	5.5 ± 1.2^a^	4.9 ± 1.2^a^
eRWC.H.o	7.2 ± 0.7	7.2 ± 0.8	7.1 ± 0.8	1.8 ± 0.7	6.1 ± 0.3^a^	5.7 ± 0.3^a^	3.3 ± 1.9^ab^	3.6 ± 2.3^ab^
eRWC.HS.y	8.4 ± 1	7.7 ± 1.4	7.1 ± 1.8	1.2 ± 1.6	3.3 ± 0.3^ab^	2.6 ± 2.3^ab^	3 ± 0.4^ab^	1.6 ± 1.4^ab^
eRWC.HS.o	7.2 ± 0.7	7 ± 0.5	7.2 ± 1.1	1.1 ± 1.2	4.1 ± 1.9^ab^	4.3 ± 2.1^ab^	1.5 ± 1.1^b^	2.1 ± 1.9^ab^

(A) RM, RM.H and eRMs produced. Within each microbial group, significant differences (α = 0.05) are marked with * for the comparisons RH-eRM, RM.H-eRM.H and RM.H-eRM.HS. The significant difference between the three eRMs is instead reported using different letters. (B) NWC and eRWCs: within each microbial group, values with different letters are significantly different. Refer to Figure 1 for samples’ abbreviation.

The enrichment step significantly increased all the microbial group concentrations in all the eRMs produced, except for facultative heterofermentative (FH) lactobacilli and yeast and molds, which were not significantly different between RM.H and eRM.HS (Table 2). The eRM showed the highest LAB concentrations (9.1 ± 0.2 log CFU/mL for both lactobacilli and streptococci), while the heat treatment and the NaCl addition inhibited the LAB growth. Streptococci concentration was not significantly different between eRM.H and eRM.HS, indicating that the NaCl addition did not influence their growth during the enrichment. The NaCl addition was instead effective in reducing enterobacteriaceae growth in eRM.HS. Staphylococci were not inhibited either by heat or salt.

The final eRWCs and the NWC showed more variable results in different microbial groups as showed by the higher SD (Table 2). As expected, viable streptococci decreased in the old cultures (eRWC.o, streptococci = 6.5 ± 0.2 log CFU/mL; mean ± SD), whereas the young cultures contained higher bacterial counts, with the highest count of lactobacilli in eRWC.y at 7.9 ± 0.2 log CFU/mL. Significant differences were found in the concentration of enterococci (eRWC.H.o vs. NWC), yeast and molds (eRWC.H.o, eRWC.o vs. NWC), staphylococci (eRWC.HS.o, NWC vs. eRWC.H.y), and enterobacteriaceae (eRWC.H.y, eRWC.o vs. NWC).

No clostridia were detected in any sample.

#### 3.1.2. Next-generation sequencing analysis

After the raw sequences trimming, a total of 557 ASVs were identified in the RM, eRM, NWC and eRWC samples, assigned to 274 species. Table 3 shows the alpha diversity of all the samples, measured with different indices (Thukral, 2017). The Chao1 and Shannon indices were used to define the microbial richness and diversity, respectively. The RM showed the highest microbial richness and diversity, although with clear differences between replicates. On the other hand, NWC was the sample that showed the least variability between the three replicates, but it had low values of Chao1 and Shannon. Heat treatment and salting of the RM influenced the microbial richness and diversity of the produced eRMs. This effect was less evident in the eRWCs, which all showed a similar species richness independently from the type of eRM mixed with the NWC for their production. The further incubation to produce old eRWCs increased the microbial diversity on average.

**Table 3:** Alpha diversity parameters of RM, eRMs, NWC, and eRWCs.

Sample	Repl.	Observed	Chao1	ACE	Shannon	Simpson	InvSimpson	Fisher
RM	1	187.0	187.3 ± 0.9	187.5 ± 5.5	3.6	0.9	11.0	28.5
	2	178.0	178 ± 0	178 ± 2.2	3.4	0.9	9.3	21.1
	3	125.0	125 ± 0.5	125.2 ± 4.3	2.3	0.8	4.6	14.0
eRM	1	33.0	33 ± 0.2	33.4 ± 2.3	1.8	0.8	4.1	3.7
	2	25.0	25 ± 0.5	25.2 ± 2.2	0.5	0.2	1.2	2.6
	3	31.0	31 ± 0.5	31.5 ± 1.6	0.4	0.1	1.1	3.1
eRM.H	1	27.0	27 ± 0	27 ± 1.6	2.1	0.8	5.8	3.0
	2	34.0	34 ± 0.2	34.7 ± 1.7	2.2	0.8	5.7	3.3
	3	48.0	48 ± 0	48 ± 1	2.1	0.8	4.7	4.6
eRM.HS	1	63.0	63 ± 0	63 ± 3.8	0.8	0.3	1.5	6.4
	2	38.0	38 ± 0	38 ± 2.8	1.9	0.8	5.3	3.6
	3	40.0	40 ± 0.5	40.3 ± 2.2	0.8	0.3	1.4	3.7
NWC	1	8.0	8 ± 0	8 ± 1.2	0.2	0.1	1.1	0.7
	2	12.0	12 ± 0	12 ± 1	0.3	0.1	1.2	1.0
	3	9.0	9 ± 0	9 ± 0.9	0.2	0.1	1.1	0.8
eRWC.y	1	19.0	19 ± 0	19 ± 1.3	0.6	0.2	1.3	1.8
	2	30.0	30 ± 0.2	30.7 ± 2.2	0.6	0.3	1.4	2.8
	3	30.0	30 ± 0	30 ± 2.5	0.3	0.1	1.1	2.9
eRWC.o	1	27.0	27 ± 0	27 ± 2	1.3	0.6	2.5	2.5
	2	31.0	31 ± 0	31 ± 1.6	1.5	0.7	3.2	2.8
	3	28.0	28 ± 0	28 ± 1.9	0.4	0.1	1.2	2.7
eRWC.H.y	1	27.0	27 ± 0.2	27.6 ± 1.6	0.7	0.3	1.4	2.7
	2	24.0	25 ± 2.3	25 ± 2.2	0.4	0.2	1.2	2.2
	3	26.0	26 ± 0	26 ± 2.5	0.4	0.1	1.1	2.5
eRWC.H.o	1	29.0	29 ± 0.5	29.3 ± 2.2	1.0	0.4	1.7	2.7
	2	21.0	21 ± 0	21 ± 1.8	1.1	0.5	1.9	1.9
	3	25.0	25 ± 0.2	25.5 ± 2.1	0.5	0.2	1.2	2.4
eRWC.HS.y	1	13.0	13 ± 0	13 ± 1.3	0.3	0.1	1.1	1.1
	2	22.0	22 ± 0.1	22.7 ± 2.2	0.6	0.3	1.4	1.9
	3	18.0	19 ± 2.3	18.7 ± 2	0.3	0.1	1.1	1.7
eRWC.HS.o	1	19.0	19 ± 0	19 ± 1.3	1.1	0.6	2.4	1.7
	2	25.0	25 ± 0	25 ± 1.4	1.5	0.7	3.5	2.2
	3	23.0	23 ± 0	23 ± 1.8	0.6	0.2	1.3	2.1

Chao1 and ACE (abundance-based coverage estimators) results report ± standard error. Refer to Figure 1 for samples’ abbreviation.

**Figure 3:**
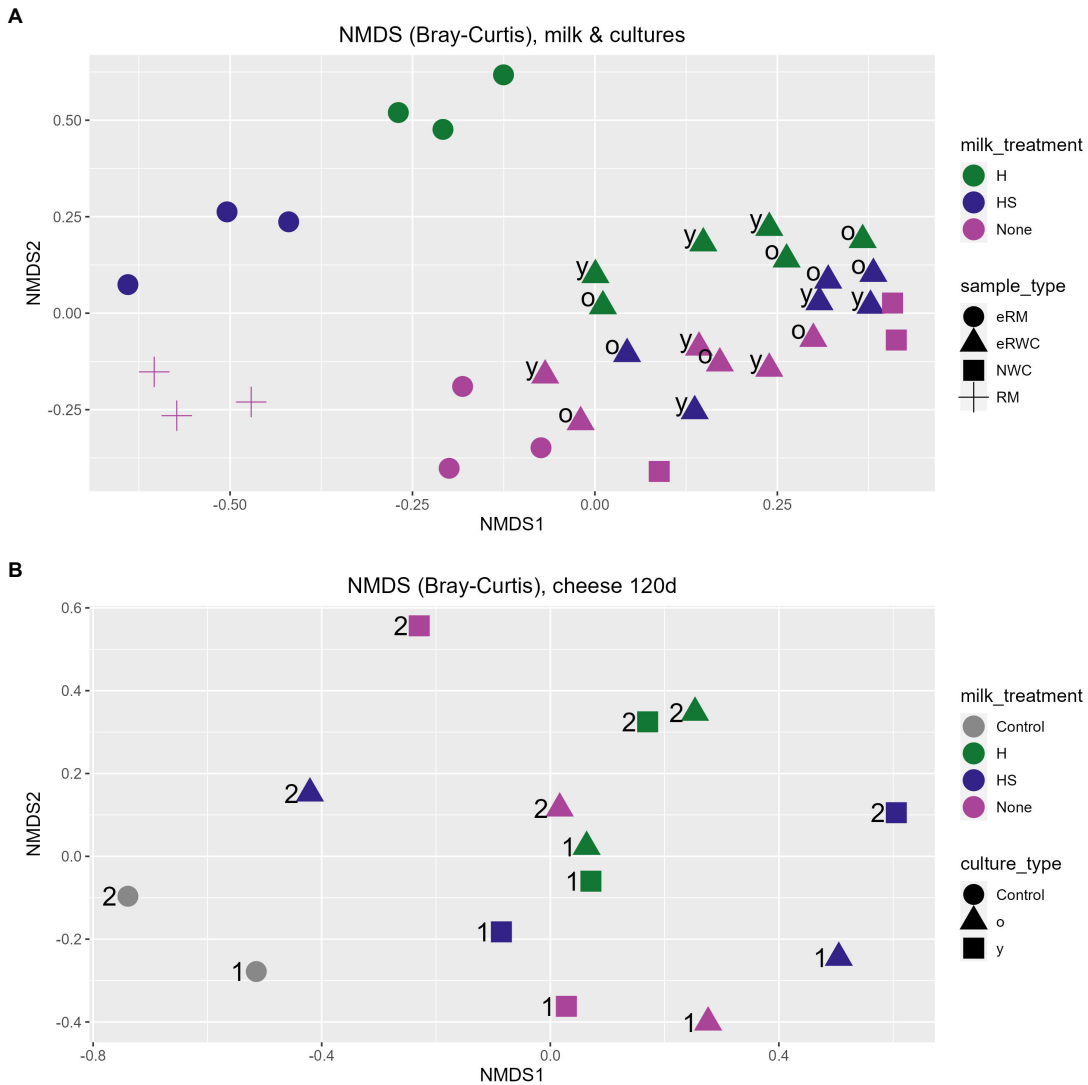
NGS results: ordination plot of non-metric multidimension scaling (NMDS) on Bray–Curtis dissimilarities. **(A)** RM, eRMs, NWC, and eRWCs.; “y” = young, “o” = old. **(B)** 120 d ripened cheese; “1” = production day 1; “2” = production day 2. Refer to Figure 1 for samples’ abbreviation.

Non-metric multidimensional scaling of Bray–Curtis dissimilarity was used to study the between-sample diversity. As shown by the ordination plot in Figure 3A, the eRWCs formed a cluster indicating that their microbiota was similar and more influenced by the NWC. While there was no clear clusterization of the eRWCs based on the “young” and “old” treatment, eRWC.H and eRWC were separated along the secondary axis of the graph, indicating that the type of eRM used for their production influenced their microbial profile, although this difference was not statistically significant according to the performed PERMANOVA test on Bray–Curtis dissimilarities. On the other hand, eRWCs’ microbiota was significantly different from the microbiota of RM and eRWCs.

**Figure 4:**
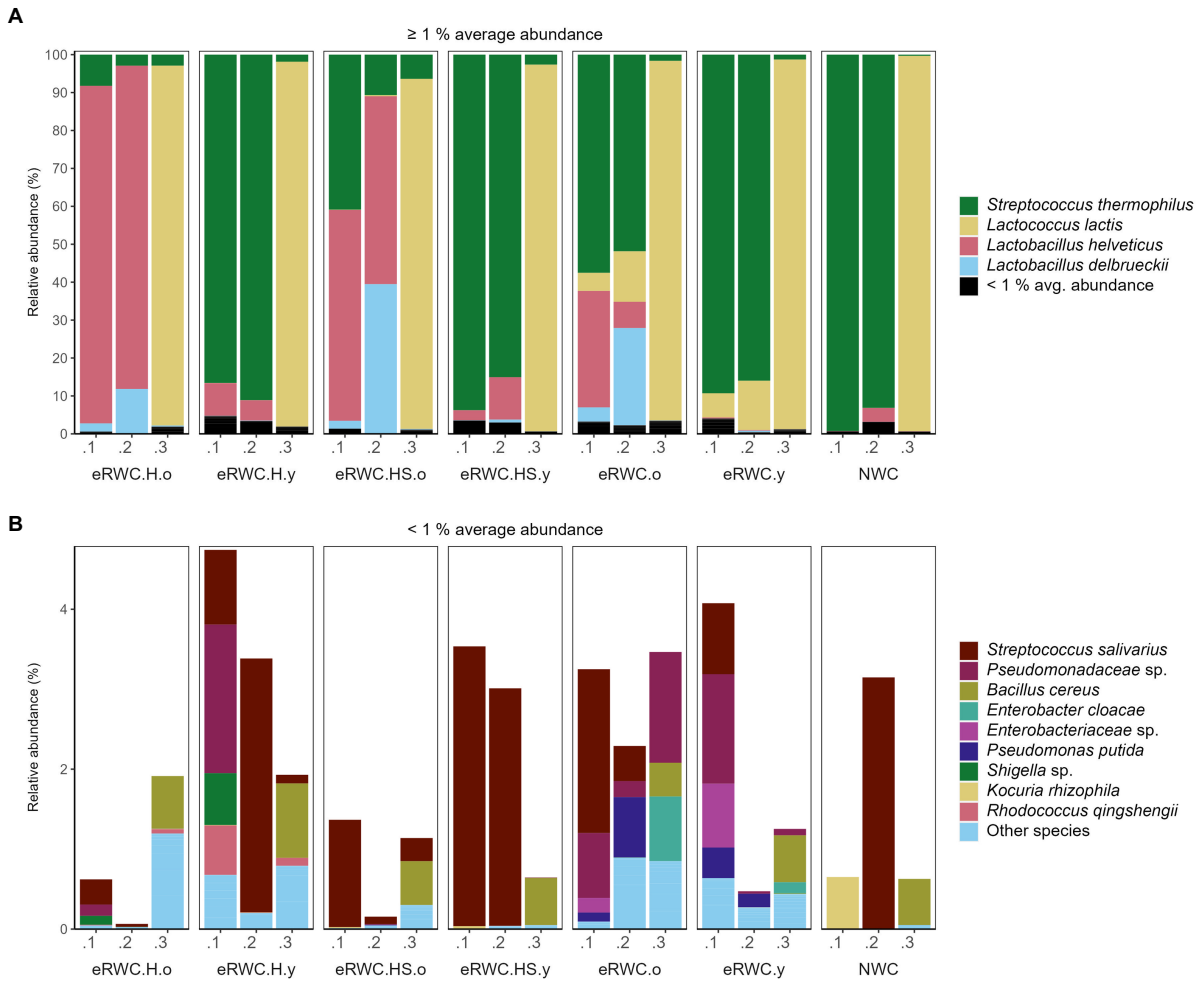
NGS results of NWC and eRWCs: stacked bar plot of species’ relative abundance (%). **(A)** Species with and average abundance ≥1%. **(B)** Species with and average abundance <1%. Refer to Figure 1 for samples’ abbreviation; the three samples’ replicates are reported.

Figures 4A,B show the dominant and subdominant species present in the NWC and the final eRWCs. Four species, namely *S. thermophilus*, *Lactococcus lactis*, *L. helveticus*, and *L. delbrueckii*, dominated the cultures, having an average relative abundance above 1%. Two replicates of NWC were primarily composed of *S. thermophilus*, at 99.3% and 93.2% abundance, respectively. Surprisingly, the third replicate contained only a small proportion of *S. thermophilus* (0.31%), whereas the main constituent was *L. lactis* at 99%, a species not commonly found in NWCs (De Filippis et al., 2014). This influenced the microbial composition of the final cultures: all the eRWC replicates 0.1 and 0.2, derived from the NWCs rich in *S. thermophilus*, showed a higher concentration of *S. thermophilus* than (when present) *L. lactis*, while the eRWC replicates 0.3 always had a concentration of *L. lactis* > 92%. Except for the replicates 0.3, all the “old” cultures were characterized by the presence and high abundance of *L. delbrueckii* and *L. helveticus*. Among the low-abundant species (average relative abundance <1%), *Streptococcus salivarus* and species belonging to the *Pseudomonaceae* family were the most present and abundant.

### 3.2. Cheese production

Cheese cross sections after 120 d of ripening are shown in Supplementary Figure S1.

The use of eRWCs slowed the acidification process during the cheese making, as shown by the higher pH values in the curd after 6 h of production (Figure 2B). After 24 h, this difference disappeared, and all the cheeses reached a pH in the target range of 4.95–5.01.

#### 3.2.1. Microbiological and chemical analysis

**Figure 5:**
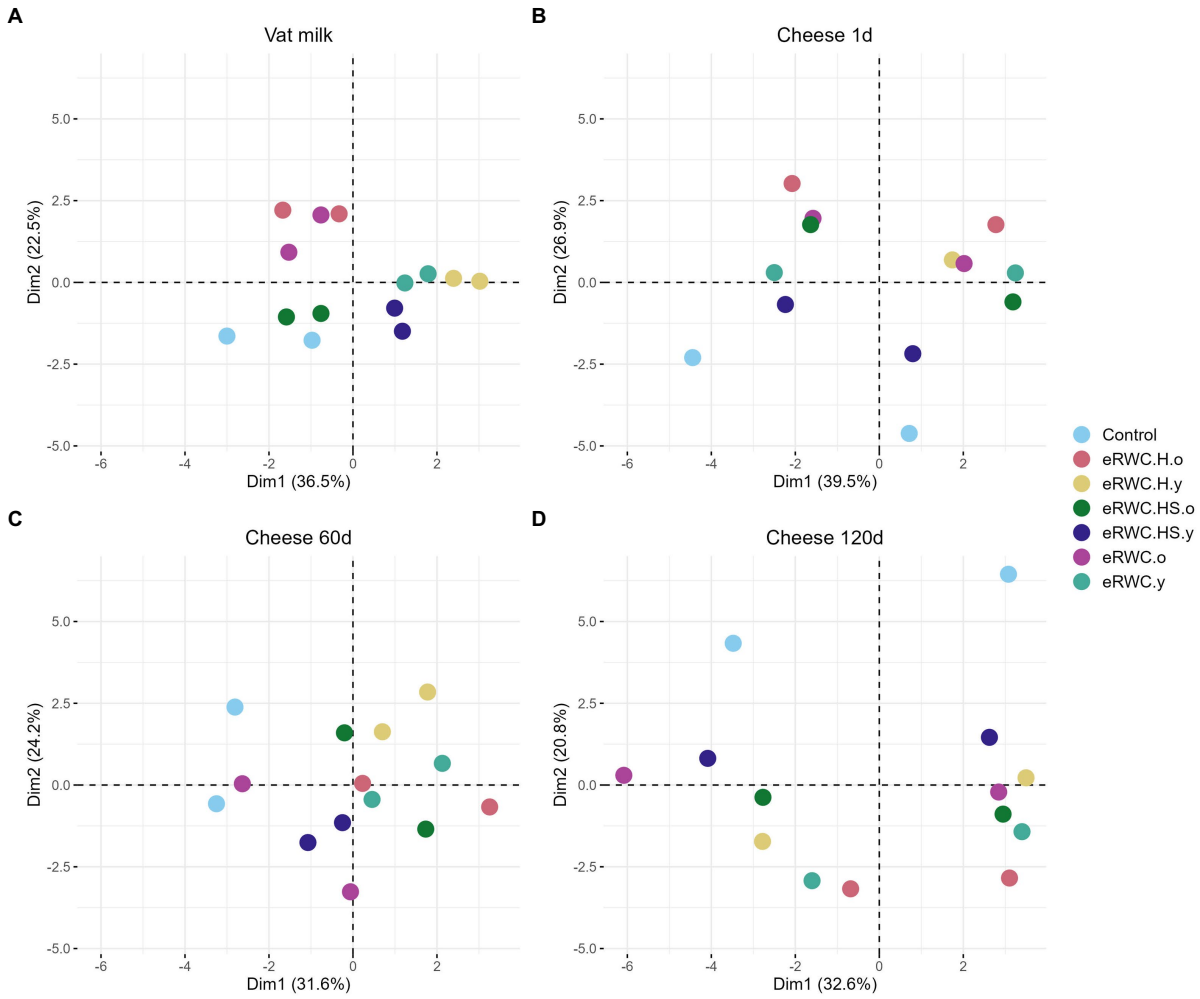
Ordination plots of principal component analysis: **(A)** vat milk, microbial features (*n* = 14); **(B)** 1 d cheese, microbial (*n* = 14) and chemical features (*n* = 7); **(C)** 60 d cheese microbial features (*n* = 14); **(D)** 120 d cheese, microbial (*n* = 14) and chemical features (*n* = 29). Refer to Figure 1 for samples’ abbreviation.

We assessed the overall similarity of analyzed samples by reducing the dimensionality of all measured parameters (*n* = 43), including both chemical (varying *n*, range 7–29) and microbiological (*n* = 14) features by principal component analysis (PCA). Ordination plots are shown in Figure 5. Raw values of chemical results of 120 d ripened cheese are reported in Supplementary Tables S2–S4. The control cheeses produced from thermized milk resulted in very different chemical and microbiological features; for this reason, in order to better evaluate the effect of the adjunct cultures on cheese properties, we excluded their results from the PCA. Constant features between the samples were removed because they did not contribute to the explanation of the samples’ similarity.

For the vat milk and the 60 d cheese, only microbiological features were analyzed. The first two principal components explained 59% of the variance in vat milk samples (Figure 5A). When produced with the same adjunct culture, milk samples shared a similar microbial profile as shown by their proximity in the ordination plot. Three clusters formed. By coloring the results by culture type, i.e., young and old (Supplementary Figure S2B), it was possible to see how the microbial profile of the vat milk was influenced by this factor. The control and the vat milk with eRWC.HS.o showed a similar microbial profile. After 60 d, the variance explained by the first two principal components, but only based on the microbiological analysis, was lower (55.8%, Figure 5C). The contribution of each variable on the first two principal components is reported in Supplementary Figure S3.

In addition to the microbiological features, different chemical features were analyzed for the cheeses one day (Supplementary Figure S4C) and 120 d (Supplementary Figure S5C) after production. The one day ripened cheeses showed two distinct clusters separated along the first component (Figure 5B), which explained 39.5% of the total variance. The factor influencing these differences was the production day (Supplementary Figure S4A), which mainly caused changes in the dry matter, dry loss, and pH of the cheese (Supplementary Figure S4C). Even if the effect was less pronounced, the use of different cultures influenced the 1 d cheese properties. This is more evident looking at the clustering of the samples on the second principal component in Figure 5B, which explained 26.9% of the variance. Being clustered at the bottom of the plot, the control cheeses showed different microbiological and chemical characteristics from the cheese produced with adjunct culture. In particular, the samples produced with eRWC.H.o were the most different from the control. The features mainly driving this distinction were the amount of D-lactic acid, citric acid, and FH lactobacilli (Supplementary Figure S4C). At the end of ripening, after 120 d, the NaCl content, together with the volatile profile and the concentration of some biogenic ammines, were the features that most contributed to the samples’ diversity (Supplementary Figure S5C). As shown in Supplementary Figure S5A, cheese samples are divided on the first component (32.6% of the total variance explained) grouping in function of the production day. Also, the adjunct culture used for cheese production influenced the cheese properties: in Figure 5D, pairs of samples are clearly separated along the second component mainly because of their content in LAP, NPN, formic acid, acetic acid, and D-lactic acid (Supplementary Figure S5C).

#### 3.2.2. Next-generation sequencing analysis

The total number of ASVs found in all the cheese samples ripened at 120 d was 62, which were assigned to 22 species. Table 4 shows the alpha diversity of the cheeses. Samples are grouped based on the type of adjunct culture used for cheese production. Looking at the Chao1 and Shannon indices of microbial richness and diversity, respectively, it is possible to see how the control cheese produced without adjunct cultures had the greatest variability between the two production replicates, while the use of eRWCs limited this variability. The cheese with the highest species richness and diversity were those produced with eRWC.H.y and eRWC.H.o, respectively. The cheese produced with eRWC.y showed lower Chao1 and Shannon values, more similar to the control cheese.

**Table 4:** Alpha diversity parameters of 120 d ripened cheese produced with different adjunct cultures.

Adjunct culture	Prod. day	Observed	Chao1	ACE	Shannon	Simpson	InvSimpson	Fisher
Control	1	25.0	25 ± 0	25 ± 2	1.5	0.6	2.8	2.5
	2	18.0	18 ± 0	18 ± 1.6	1.1	0.5	1.9	1.7
eRWC.y	1	23.0	23 ± 0.5	–	1.3	0.6	2.8	2.1
	2	20.0	20 ± 0	20 ± 1.8	1.2	0.5	2.2	1.9
eRWC.o	1	24.0	24 ± 0	24 ± 1.6	1.4	0.7	3.1	2.2
	2	27.0	27 ± 0	27 ± 1.6	1.3	0.6	2.6	2.6
eRWC.H.y	1	27.0	27 ± 0.5	27.5 ± 1.4	1.5	0.7	3.5	2.6
	2	29.0	29 ± 0	29 ± 1.9	1.7	0.7	3.9	2.7
eRWC.H.o	1	28.0	28 ± 0.5	28.3 ± 1.9	1.5	0.7	3.3	2.7
	2	27.0	27 ± 0	27 ± 1.4	1.7	0.7	3.9	2.6
eRWC.HS.y	1	25.0	25 ± 0.5	25.6 ± 1.4	1.5	0.7	3.6	2.3
	2	24.0	24 ± 0	24 ± 1.6	1.5	0.7	3.8	2.2
eRWC.HS.o	1	27.0	27 ± 0.2	28.1 ± 2	1.4	0.7	3.2	2.6
	2	24.0	24 ± 0	24 ± 1.8	1.4	0.6	2.8	2.3

Chao1 and ACE (abundance-based coverage estimators) results report ± standard error. Refer to Figure 1 for samples’ abbreviation.

Figure 3B shows the difference in the microbiota between the ripened cheese based on Bray–Curtis dissimilarity. Cheeses produced with eRWC.H.y and eRWC.H.o are close to each other, meaning that they had a similar microbiota and were less influenced by the y and o incubation. This was different for cheese produced with eRWC and eRWC.HS. Overall, the samples did not show a distinct microbiota influenced by the different cultures, but the experimental cheeses showed a significant different microbiota in comparison with the control cheeses produced with no adjunct culture. On the other hand, the cheeses microbial profiles were also influenced by the production day, i.e., by the different raw milk used for cheese making, as showed by the samples separation along the second dimension in Figure 3B.

**Figure 6:**
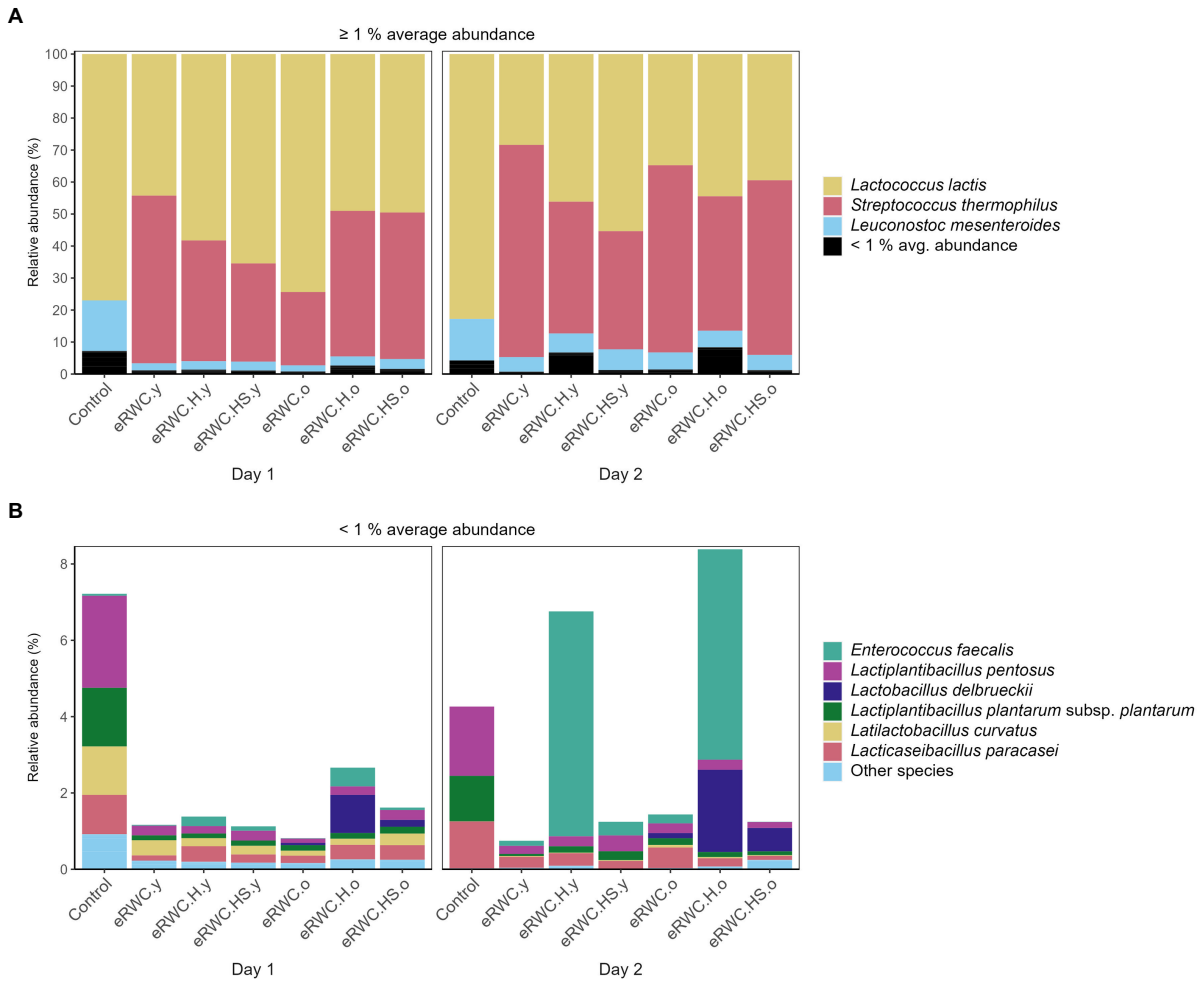
NGS results of 120 d ripened cheese produced with the different eRWCs: stacked bar plot of species’ relative abundance (%). **(A)** Species with and average abundance ≥1%. **(B)** Species with and average abundance <1%. Refer to Figure 1 for samples’ abbreviation; the two samples’ replicates (day 1 and day 2) are reported.

Figures 6A,B show the dominant and subdominant species found in the ripened cheeses. *S. thermophilus*, *L. lactis*, and *Leuconostoc mesenteroides* were the only three species present with a relative abundance above 1% on average. *S. thermophilus* was always present in the cheeses produced with the adjunct culture, but not in the control. Among the subdominant species, *L. delbrueckii* was present with higher relative abundance in cheese produced with “old” eRWCs, while in the other cheeses it was classified as *Other species* having a maximum relative abundance <0.6%. The cheeses produced with eRWC.H.y and eRWC.H.o had a particular presence of *Enterococcus faecalis*, with a relative abundance of 5.89% and 5.51%, respectively.

## 4. Discussion

As a fermented food, cheese relies on microorganisms for its production. Microorganisms are often added in the form of cultures during the cheese making mainly to drive the acidification (starter cultures) or the ripening process (starter and adjunct cultures). While starter cultures are produced both at the laboratory or industrial (mixed defined-strain cultures), and artisanal (natural undefined strain culture) level, adjunct cultures with NSLAB are only commercialized as mixed defined-strain cultures. NSLAB are naturally present in raw milk, however, although some species may have very low abundance, since LAB are just a subdominant part of the raw milk microbiota (Bettera et al., 2023). This makes their selection and isolation difficult. Strategies to overcome this limit could be the use of selective/elective growth media (Coeuret et al., 2003; Neviani et al., 2009) and/or the spontaneous fermentation of raw milk with the application of selective conditions, as already applied for the isolation of potentially probiotic yeasts and LAB (Galli et al., 2022a,b).

### 4.1. Natural adjunct culture microbiota

In this study, we performed a spontaneous fermentation of raw milk with the aim of producing a natural adjunct culture rich in NSLAB. The strategy was to apply the conditions that are known to promote the selection of NSLAB of our interest and inhibit the rest of the microbiota, as it happens in raw milk, cooked, long-ripened cheese varieties (Gatti et al., 2014; Bottari et al., 2018). Low values of pH, curd cooking, NaCl, low temperature, lack of lactose and microbial competition are factors that make the cheese environment hostile during ripening. NSLAB are subjected to these stress conditions, and their adaptation responses are efficient (Gobbetti et al., 2015).

We applied these conditions to raw milk to produce the eRWCs (Figure 1). A similar approach was used by Bancalari and colleagues, who subjected Parmigiano Reggiano raw milk samples to spontaneous fermentation for 4 months at 8°C in order to isolate potential aroma-producing *Lacticaseibacillus* strains (Bancalari et al., 2017).

The raw milk enrichment step increased the LAB concentration (Table 2) and decreased the overall microbial richness and diversity (Table 3), although differently between eRM, eRM.H and eRM.HS (Figure 3A). eRM in fact selected an aciduric microbiota due to its lower pH (Table 1) with values closer to a *lattoinnesto* (Parente et al., 2017), while eRM.H and eRM.HS were most likely composed of heat- and salt-resistant microorganisms.

We then mixed 10 mL of eRMs in 90 mL of NWC and incubated for 6 and 22 h to apply further acid stress. The LAB viable count was not significantly different between the eRWCs, ranging from 6.5 to 7.9 log CFU/mL which is comparable to LAB concentrations found in natural milk cultures (Parente et al., 2017) or natural whey cultures (Gatti et al., 2014). However, the eRWCs harbored higher microbial richness and diversity than NWC (Table 3), especially in the old cultures where lower pH selected *L. helveticus* and the more acid-resistant *L. delbrueckii* (De Angelis and Gobbetti, 2004).

The applied pre-treatments allowed to have adjunct cultures with diverse microbiota, which were tested in cheese making trials.

### 4.2. Influence of enriched raw milk whey cultures on cheese features

The addition of 0.5% eRWC was enough to influence the vat milk microbial profile (Figure 5A). Old and young eRWC samples clearly differed from each other and the control mainly because of their streptococci and yeast and mold concentrations (Supplementary Figure S2B). The use of eRWCs slowed curd acidification in the first hours of cheese making (Figure 2B). Although the acidifying performances are known to be strains-dependent (Bancalari et al., 2016), this was surprising since one of the common drawbacks of adjunct cultures is the curd over-acidification due to lactose fermentation in addition to primary starters, which is the reason why adjunct strains are usually attenuated (Gobbetti et al., 2015). Competition for nutrients between SLAB and NSLAB could be a possible explanation supported by the higher D-lactate detected in cheese with adjunct culture (samples distribution on the second dimension, Supplementary Figure S4B), which is known to be produced from NSLAB by fermentation of residual lactose or by isomerization of L-lactate (McSweeney et al., 2017; Blaya et al., 2018). However, the pH after 24 h settled to equal values for all the cheeses.

The cheese features after one day were only partially influenced by the microbiota of the eRWCs. The different amounts of D-lactic acid, citric acid, and FH lactobacilli separated the experimental cheeses along the secondary axis of the PCA (Figure 5B). In particular, the cheese produced with eRWC.H.o, the culture with the highest concentration of *L. helveticus* (Figure 4A), was the most distant from the control. On the other hand, the cheese chemical and microbiological features were mainly influenced by the production day, therefore most likely by the raw milk characteristics (Supplementary Figure S4A). The same was noticed after 120 d of ripening. Although the use of diverse eRWCs contributed in some cases to producing cheese with richer and more diverse microbiota, the final cheese features were more influenced by the production day (Supplementary Figure S5A). Variability in cheese characteristics arising from the diverse raw milk microbiota evolution during ripening is known to occur, even if the same technology is applied and starter cultures are used to drive the fermentation. Such an example was reported for Grana Padano PDO (Lazzi et al., 2016) and Cantal PDO (Frétin et al., 2018) cheeses. This result could also be caused by the cheese variety analyzed in this study. Vacherin Fribourgeois PDO is in fact a surface smear-ripened cheese. Although the richer rind microbiota of this cheese variety is known to be different from the core (Dugat-Bony et al., 2016), the surface microbiota strongly affects the final cheese characteristic. This could have masked the effect of the adjunct culture used in our trials. Perspective for future studies could be the control of the entire cheese microbiota including the rind. Furthermore, testing the eRWCs with different cheese varieties (i.e., different technological stress) could reveal different dynamics in the microbiota evolution and possibly a stronger effect of the adjunct culture on the ripened cheese (Figure 6).

This study provides new insights into the possibility to enrich the raw milk microbiota for the production of cheese. Our results showed that the applied raw milk enrichment protocols were able to increase the concentration of autochthonous LAB, and that the combination of heating and osmotic stresses at this step are effective in control the presence of some undesired microbial groups. This allowed us to produce natural adjunct cultures harboring diverse microbiota. Chemical and microbiological results suggested that this microbial diversity influenced the early stages of cheese making, but its effect decreased over time during ripening, showing an inferior effect than that of raw milk microbiota. More research is needed to optimize the culture production, including testing different treatments that could more specifically select desired NSLAB present in raw milk, or try different ratios of eRM-NWC mixing since our results showed a strong influence of the NWC on the eRWC microbiota. The optimization of such a tool could be an alternative to the practice of isolating, geno-pheno-typing, and formulating mixed-defined-strain adjunct cultures that require knowledge and facilities not always available for artisanal cheese makers.

## Data availability statement

The data presented in the study are deposited in the “eRWC” repository, doi: 10.5281/zenodo.7736671. The NGS data presented in the study are deposited in the Sequence Read Archive (SRA), BioProject ID: PRJNA937653.

## Author contributions

LB, MD, RS, and H-PB contributed to conception and design of the study. LB, MD, and HB performed the experiments. LB and MD performed the data curation, elaboration, and statistical analysis. LB wrote the first draft of the manuscript. MG, RS, and H-PB acquired the fundings. All authors contributed to the article and approved the submitted version.

## Funding

LB received a travel grant provided by the University of Parma. All other expenses were covered by the University of Parma and Agroscope. Open access funding was provided by Agroscope.

## Conflict of interest

The authors declare that the research was conducted in the absence of any commercial or financial relationships that could be construed as a potential conflict of interest.

## Publisher’s note

All claims expressed in this article are solely those of the authors and do not necessarily represent those of their affiliated organizations, or those of the publisher, the editors and the reviewers. Any product that may be evaluated in this article, or claim that may be made by its manufacturer, is not guaranteed or endorsed by the publisher.
