# Supplementary material for: Selective enrichment of the raw milk microbiota in cheese production: Concept of a natural adjunct milk culture
Source: Front Microbiol. 2023 Apr 26;14:1154508. doi: 10.3389/fmicb.2023.1154508 (PMC10169670; doi:10.3389/fmicb.2023.1154508)
Supplement: Supplementary file 1 [file Data_Sheet_1.docx]

Supplementary Material

**Supplementary Table 1.** Microbial groups analyzed for viable counts and methods used.

| Microbial group | Incubation conditions | Atmosphere conditions | Growth medium | Supplier |
| --- | --- | --- | --- | --- |
| Total aerobic mesophilic (TAM) | 30 °C for 2 days | aerobic | Standard Methods Agar with Casein | -BD (Franklin Lakes, NJ, U.S.) -Merck (Casein peptone) (Darmstadt, Germany) |
| Streptococci | 37 °C for 2 days | aerobic | M 17 agar  + D(+) Glucose (Terzaghi and Sandine, 1975) | Merck |
| Lactobacilli | 30 °C for 2 days | anaerobic | MRS-lactose agar (5M lactic acid) (De Man et al., 1960) | -Biolife (Milan, Italy)  -Oxoid (Agar No 2.) (Waltham, MA, U.S.) |
| Facultative heterofermentative lactobacilli (FHL) | 30 °C for 3-4 days | anaerobic | FH agar (Isolini et al., 1990) | -Biolife |
| Propionibacteria | 30 °C for 7 days | anaerobic | Lactate agar | -Merck (Lactate solution, casein peptone)  -BBL (Yeast extract)  -Oxoid (Agar No 2) |
| Enterococci | 37 °C for 2 days | aerobic | Kanamycin Esculin Azide agar (Mossel et al., 1978) | Merck |
| Yeasts and molds | 30 °C for 3-4 days | aerobic | Phytone-Yeast-Extract Agar | BBL |
| Staphylococci | 37 °C for 2 days | aerobic | Mannitol salt phenol-red agar | Biolife |
| Enterobacteriaceae | 37 °C for 2 days | aerobic | MacConkey agar | Oxoid |

**Supplementary Table 2.** Biogenic ammines (mg/kg) and free amino acids (mmol/kg) content in 120 d ripened cheese. OPA = ophthaldialdehyde. Refer to **Figure** **1** in the article for samples’ abbreviation.

| Parameter | Adjunct culture | | | | | | | | | | | | | |
| --- | --- | --- | --- | --- | --- | --- | --- | --- | --- | --- | --- | --- | --- | --- |
|  | Control | | eRWC.y | | eRWC.o | | eRWC.H.y | | eRWC.H.o | | eRWC.HS.y | | eRWC.HS.o | |
|  | Production day | | | | | | | | | | | | | |
|  | 1 | 2 | 1 | 2 | 1 | 2 | 1 | 2 | 1 | 2 | 1 | 2 | 1 | 2 |
| Tot. biogenic.amine | 287.5 | 77.0 | 398.0 | 159.0 | 391.0 | 157.0 | 481.0 | 200.0 | 531.0 | 158.0 | 391.0 | 133.0 | 421.0 | 117.0 |
| Cadaverin | 150.5 | 24.7 | 172.0 | 22.6 | 191.0 | 21.9 | 200.0 | 19.7 | 245.0 | 19.0 | 204.0 | 27.5 | 223.0 | 16.8 |
| Histamin | 0.0 | 0.0 | 0.0 | 0.0 | 0.0 | 0.0 | 0.0 | 0.0 | 0.0 | 0.0 | 0.0 | 0.0 | 0.0 | 0.0 |
| Isopentylamin | 0.0 | 0.0 | 0.0 | 0.0 | 0.0 | 0.0 | 0.0 | 0.0 | 0.0 | 0.0 | 0.0 | 0.0 | 0.0 | 0.0 |
| Phenylethylamine | 20.5 | 7.9 | 32.4 | 33.3 | 23.1 | 21.6 | 45.6 | 39.4 | 45.6 | 27.0 | 17.0 | 19.6 | 16.6 | 16.6 |
| Putrescin | 36.6 | 0.0 | 94.3 | 0.0 | 88.7 | 0.0 | 106.0 | 0.0 | 119.0 | 0.0 | 88.0 | 0.0 | 102.0 | 0.0 |
| Spermidin | 0.0 | 0.0 | 0.0 | 0.0 | 0.0 | 0.0 | 0.0 | 0.0 | 0.0 | 0.0 | 0.0 | 0.0 | 0.0 | 0.0 |
| Spermin | 0.0 | 0.0 | 0.0 | 0.0 | 0.0 | 0.0 | 0.0 | 0.0 | 0.0 | 0.0 | 0.0 | 0.0 | 0.0 | 0.0 |
| Tryptamine | 0.0 | 0.0 | 0.0 | 0.0 | 0.0 | 0.0 | 0.0 | 0.0 | 0.0 | 0.0 | 0.0 | 0.0 | 0.0 | 0.0 |
| Tyramin | 79.7 | 44.5 | 99.6 | 103.0 | 88.0 | 114.0 | 128.0 | 141.0 | 120.0 | 112.0 | 81.9 | 85.9 | 79.7 | 83.8 |
| OPA-value (mmol/kg) | 184.4 | 210.4 | 151.8 | 152.8 | 159.4 | 172.0 | 154.4 | 167.7 | 158.2 | 178.3 | 153.6 | 153.0 | 154.1 | 169.8 |

**Supplementary Table 3.** Chemical composition of 120 d ripened cheese. LAP = L-leucine aminopeptidase; NPN = non-protein nitrogen; WSN = water-soluble nitrogen; TN = total nitrogen. Refer to **Figure** **1** in the article for samples’ abbreviation.

| Parameter | Adjunct culture | | | | | | | | | | | | | |
| --- | --- | --- | --- | --- | --- | --- | --- | --- | --- | --- | --- | --- | --- | --- |
|  | Control | | eRWC.y | | eRWC.o | | eRWC.H.y | | eRWC.H.o | | eRWC.HS.y | | eRWC.HS.o | |
|  | Production day | | | | | | | | | | | | | |
|  | 1 | 2 | 1 | 2 | 1 | 2 | 1 | 2 | 1 | 2 | 1 | 2 | 1 | 2 |
| NaCl (g/kg) | 14.9 | 21 | 14.4 | 20.6 | 14.6 | 22.2 | 14.9 | 20 | 15.3 | 20 | 16.2 | 20.7 | 17.2 | 21.1 |
| Citric acid (mmol/kg) | 0 | 0 | 0 | 0 | 0 | 0 | 0 | 0 | 0 | 0 | 0 | 0 | 0 | 0 |
| D-lactic acid (mmol/kg) | 29.45 | 32.9 | 46.8 | 47.8 | 48.8 | 45.7 | 45.7 | 46.6 | 42.6 | 45.1 | 48.1 | 47.7 | 43.3 | 47 |
| L-lactic acid (mmol/kg) | 78 | 66.15 | 65.5 | 56.5 | 64.1 | 53.3 | 63.5 | 56.1 | 66.7 | 67.1 | 61.8 | 52.5 | 64 | 60.6 |
| LAP (IU/kg) | 1.3 | 2.475 | 5.45 | 6.95 | 5.1 | 5.75 | 5.3 | 5.65 | 6.1 | 6.35 | 5.95 | 5.85 | 5.15 | 6.15 |
| Dry loss (g/kg) | 404.5 | 406.5 | 413 | 398 | 411 | 415 | 423 | 412 | 415 | 410 | 414 | 419 | 418 | 411 |
| Water in fat free cheese (g/kg) | 603 | 591 | 613 | 589 | 608 | 602 | 618 | 601 | 605 | 600 | 614 | 607 | 616 | 600 |
| Fat (g/kg) | 329.5 | 312 | 326 | 324 | 324 | 311 | 316 | 315 | 314 | 317 | 326 | 310 | 321 | 315 |
| FDM (g/kg) | 553.5 | 525.5 | 555 | 538 | 550 | 532 | 548 | 536 | 537 | 537 | 556 | 534 | 552 | 535 |
| NPN (g/kg) | 6.82 | 6.89 | 6.2 | 5.55 | 6.23 | 5.87 | 6.24 | 5.92 | 6.21 | 6.02 | 6.35 | 5.97 | 6.04 | 5.94 |
| WSN (g/kg) | 9.925 | 12.65 | 11.3 | 12.5 | 10.4 | 16 | 11.3 | 12.7 | 11.5 | 12 | 10.4 | 10.7 | 12.7 | 11.9 |
| TN (g/kg) | 36.25 | 37.2 | 35.9 | 34.8 | 35.9 | 35.8 | 35.1 | 36.5 | 36.5 | 36.5 | 34.6 | 35.7 | 34.9 | 35.7 |

**Supplementary Table 4.** Volatile carboxylic acids (VOCs; mmol/kg) of 120 d ripened cheese. Refer to **Figure** **1** in the article for samples’ abbreviation.

| Compound | Adjunct culture | | | | | | | | | | | | | |
| --- | --- | --- | --- | --- | --- | --- | --- | --- | --- | --- | --- | --- | --- | --- |
|  | Control | | eRWC.y | | eRWC.o | | eRWC.H.y | | eRWC.H.o | | eRWC.HS.y | | eRWC.HS.o | |
|  | Production day | | | | | | | | | | | | | |
|  | 1 | 2 | 1 | 2 | 1 | 2 | 1 | 2 | 1 | 2 | 1 | 2 | 1 | 2 |
| Acetic acid | 15.2 | 16.5 | 16.5 | 14.5 | 16.0 | 19.6 | 17.7 | 16.4 | 17.1 | 13.2 | 16.0 | 18.6 | 16.7 | 17.3 |
| Butyric acid | 0.8 | 1.2 | 0.7 | 1.2 | 0.6 | 1.5 | 0.8 | 1.5 | 0.7 | 1.2 | 0.8 | 1.5 | 1.0 | 1.1 |
| Caproic acid | 0.1 | 0.2 | 0.2 | 0.2 | 0.1 | 0.2 | 0.1 | 0.1 | 0.1 | 0.1 | 0.2 | 0.2 | 0.2 | 0.1 |
| Formic acid | 3.4 | 3.8 | 3.8 | 3.9 | 3.9 | 5.0 | 4.4 | 4.5 | 3.5 | 3.5 | 4.1 | 5.0 | 3.9 | 4.4 |
| Isobutyric acid | 0.1 | 0.2 | 0.0 | 0.1 | 0.0 | 0.3 | 0.1 | 0.2 | 0.0 | 0.1 | 0.0 | 0.2 | 0.1 | 0.1 |
| Isocaproic acid | 0.0 | 0.1 | 0.0 | 0.1 | 0.0 | 0.2 | 0.0 | 0.2 | 0.0 | 0.1 | 0.0 | 0.2 | 0.0 | 0.1 |
| Isovaleric acid | 0.1 | 0.3 | 0.1 | 0.2 | 0.1 | 0.5 | 0.1 | 0.3 | 0.1 | 0.2 | 0.1 | 0.3 | 0.2 | 0.2 |
| Propionic acid | 1.3 | 0.6 | 0.5 | 0.4 | 0.5 | 0.6 | 0.8 | 0.6 | 0.8 | 0.4 | 0.8 | 0.5 | 0.9 | 0.3 |
| Tot. VOCs | 21.1 | 22.9 | 21.7 | 20.5 | 21.2 | 27.9 | 24.0 | 23.8 | 22.4 | 18.7 | 22.1 | 26.4 | 22.9 | 23.7 |


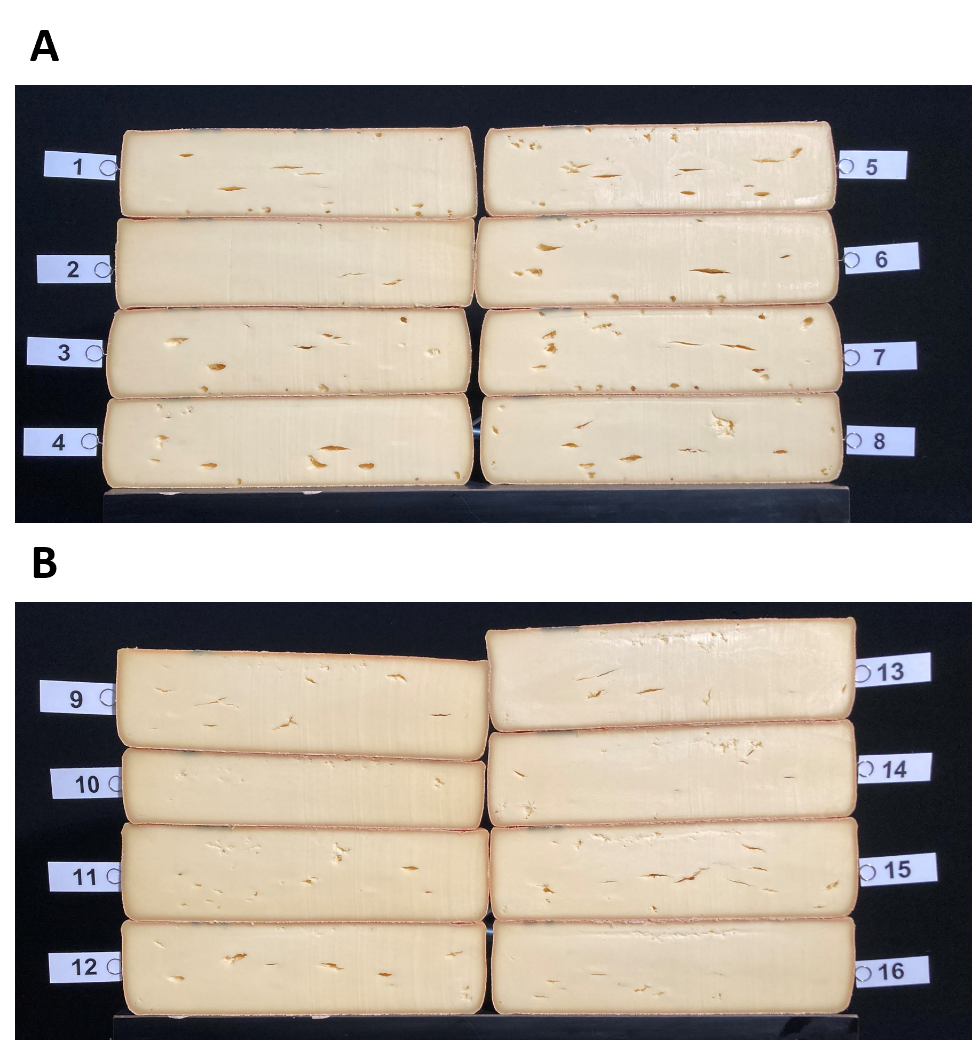


**Supplementary Figure 1.** Cheese cross sections after 120 days of ripening. **A**) First replicate; **B**) Second replicate. 1 and 9 = control, raw milk; 2 and 10 = control, thermized milk; 3 and 11 = eRWC.y; 4 and 12 = eRWC.H.y; 5 and 13 = eRWC.HS.y; 6 and 14 = eRWC.o; 7 and 15 = eRWC.H.o; 8 and 16 = eRWC.HS.o. Refer to **Figure** **1** in the article for samples’ abbreviation.


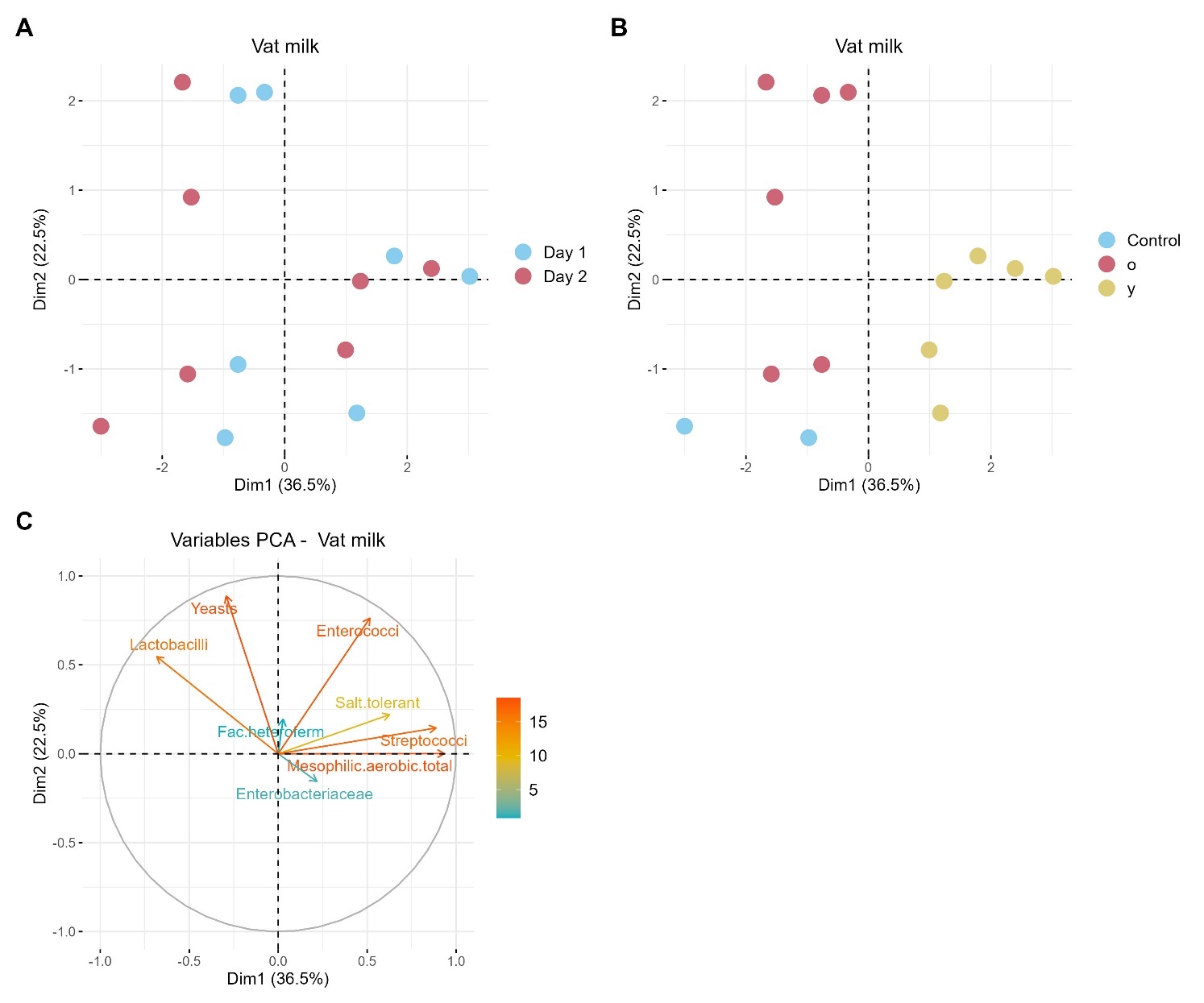


**Supplementary Figure 2.** Principal component analysis of vat milk microbial features (n = 14, before constant features removal). **A**) Ordination plot grouping by eRWC production days 1 and 2; **B**) Ordination plot grouping by eRWC treatment young (y) and old (o) (control = no use of adjunct culture); **C**) Variables contribution plot. Refer to **Figure** **1** in the article for samples’ abbreviation.


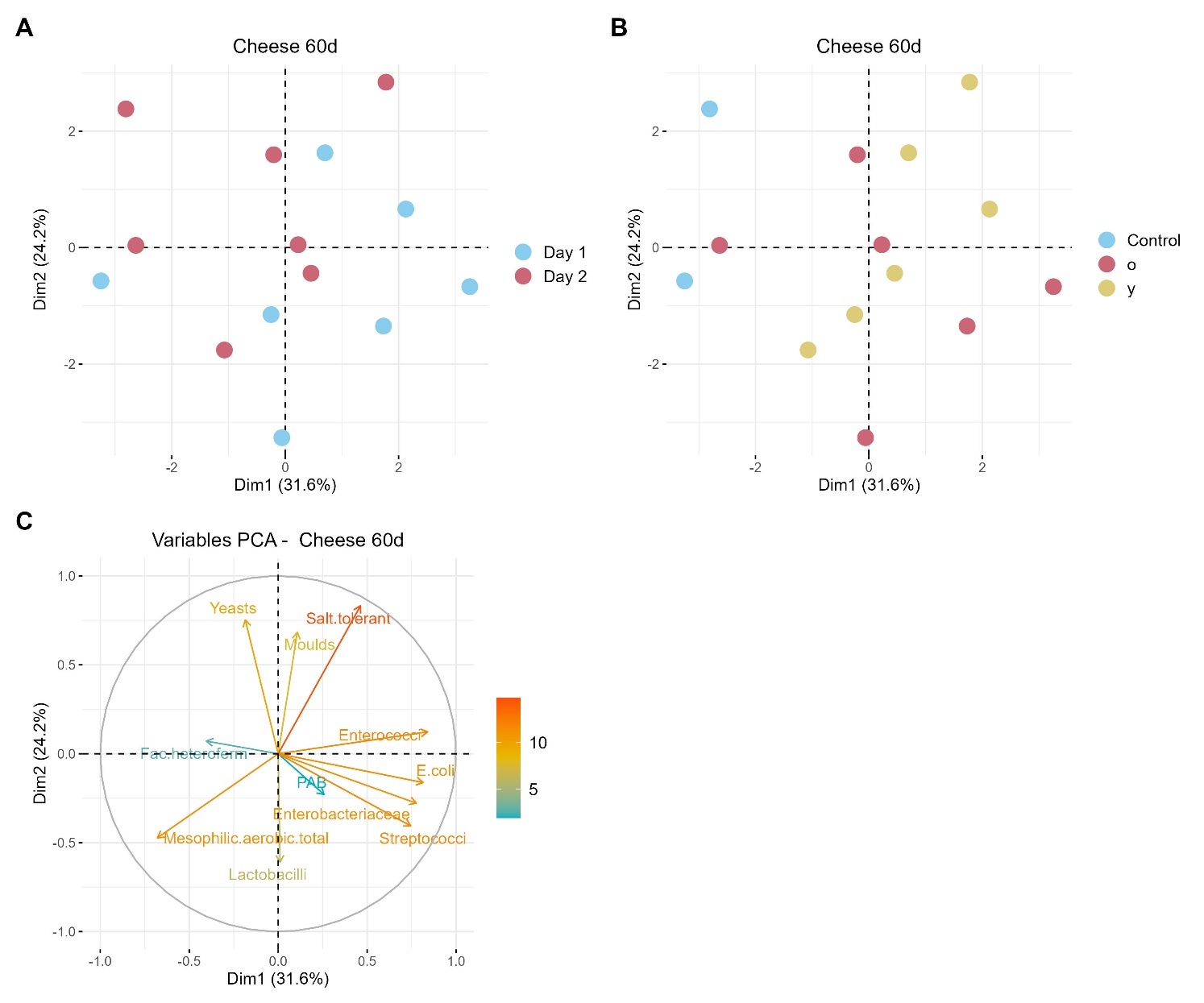


**Supplementary Figure 3.** Principal component analysis of 60 d ripened cheese microbial features (n = 14, before constant features removal). **A**) Ordination plot grouping by eRWC production days 1 and 2; **B**) Ordination plot grouping by eRWC treatment young (y) and old (o) (control = no use of adjunct culture); **C**) Variables contribution plot. Refer to **Figure** **1** in the article for samples’ abbreviation.


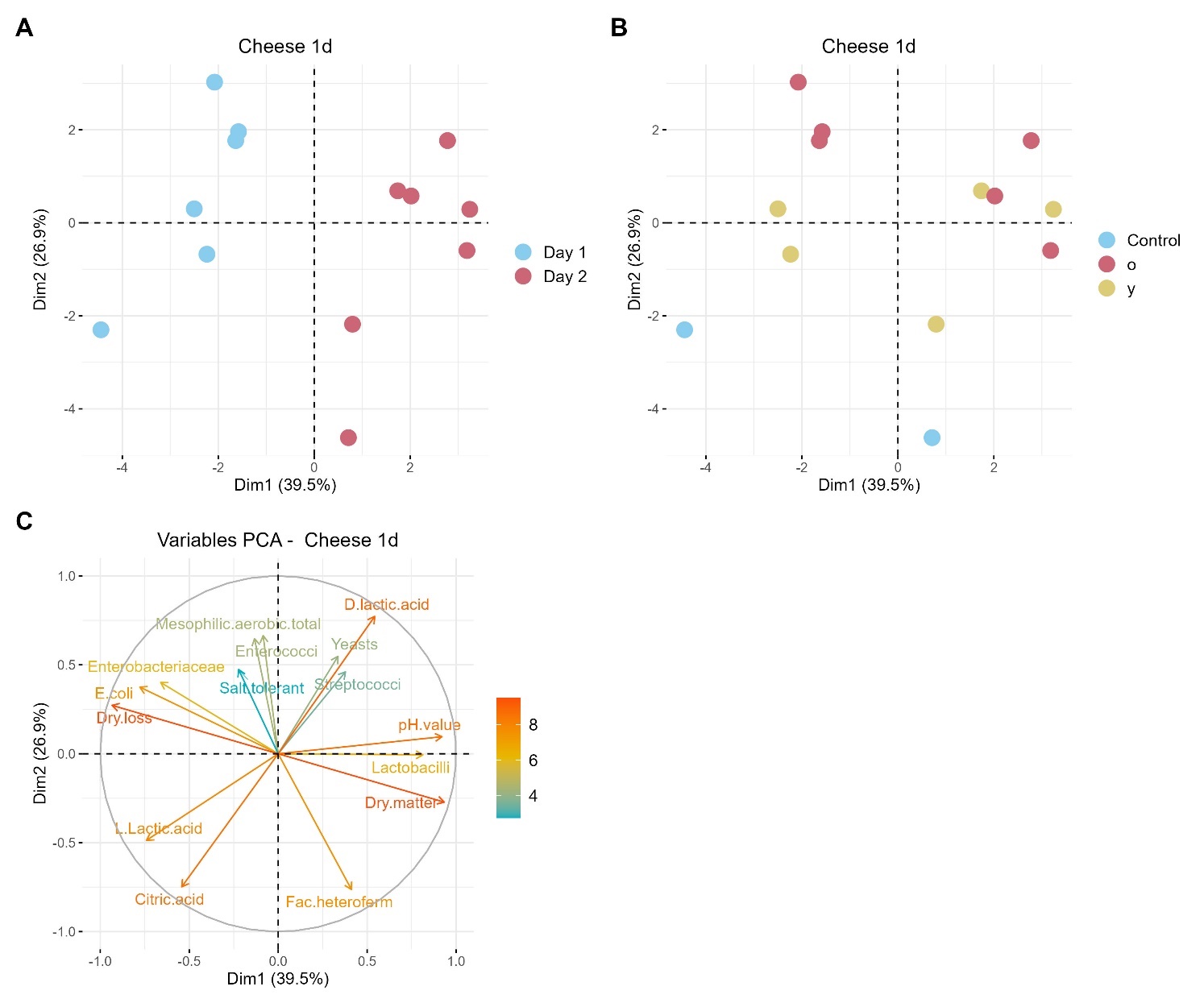


**Supplementary Figure 4.** Principal component analysis of 1 d ripened cheese microbial (n = 14, before constant features removal) and chemical (n = 7, before constant features removal) features. **A**) Ordination plot grouping by eRWC production days 1 and 2; **B**) Ordination plot grouping by eRWC treatment young (y) and old (o) (control = no use of adjunct culture); **C**) Variables contribution plot. Refer to **Figure** **1** in the article for samples’ abbreviation.


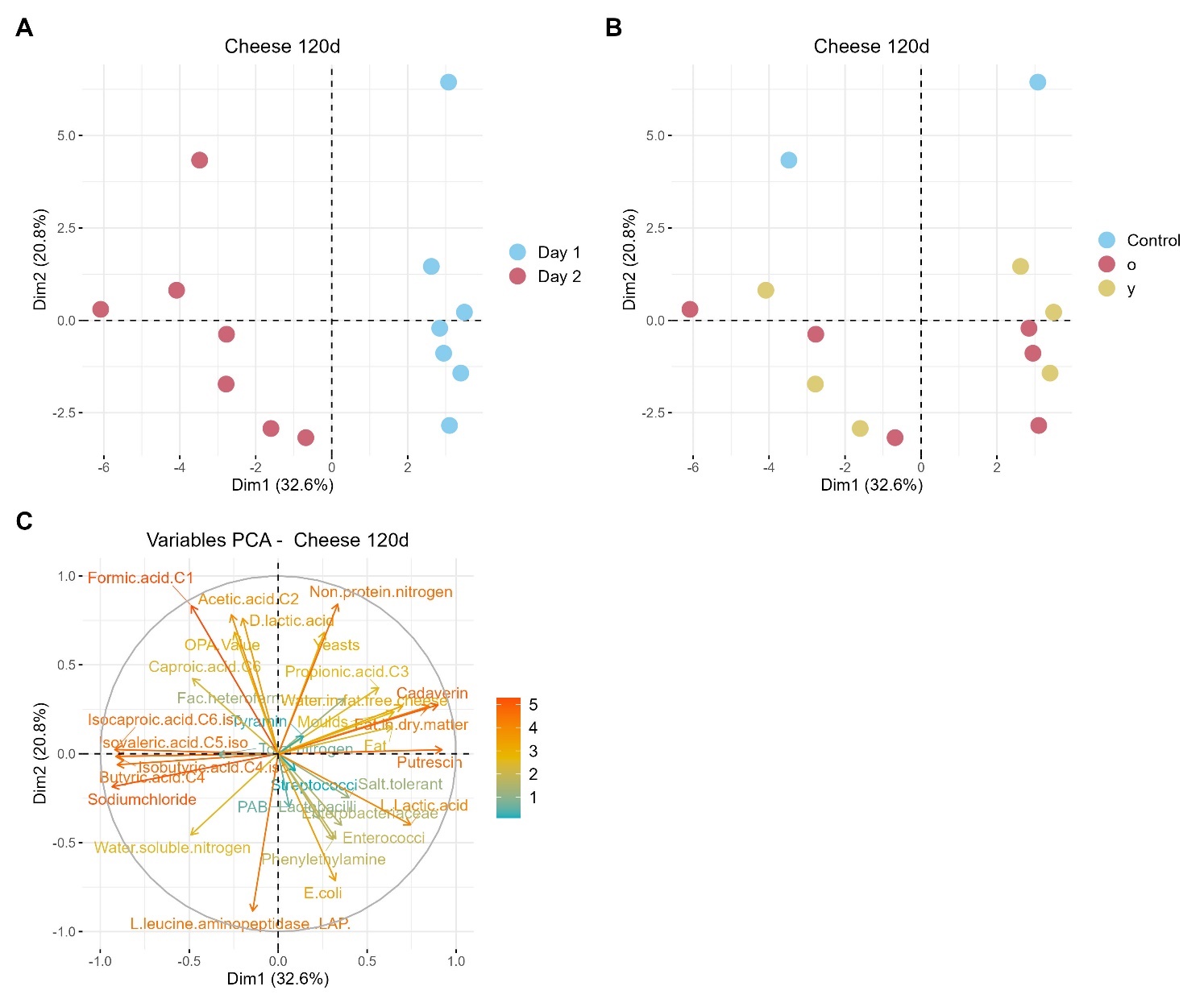


**Supplementary Figure 5.** Principal component analysis of 120 d ripened cheese microbial (n = 14, before constant features removal) and chemical (n = 29, before constant features removal) features. **A**) Ordination plot grouping by eRWC production days 1 and 2; **B**) Ordination plot grouping by eRWC treatment young (y) and old (o) (control = no use of adjunct culture); **C**) Variables contribution plot. Refer to **Figure** **1** in the article for samples’ abbreviation.
